# Plasma Metabolome Alterations Associated with Extrauterine Growth Restriction

**DOI:** 10.3390/nu12041188

**Published:** 2020-04-23

**Authors:** Danuta Dudzik, Isabel Iglesias Platas, Montserrat Izquierdo Renau, Carla Balcells Esponera, Beatriz del Rey Hurtado de Mendoza, Carles Lerin, Marta Ramón-Krauel, Coral Barbas

**Affiliations:** 1 Centro deMetabolómica y Bioanálisis, Facultad de Farmacia, Universidad San Pablo-CEU, CEU Universities, Urbanización Montepríncipe, Boadilla del Monte, 28660 Madrid, Spain or; 2 Department of Biopharmaceutics and Pharmacodynamics, Faculty of Pharmacy, Medical University of Gdansk, 80-416 Gdańsk, Poland; 3 Neonatal Unit, BCNatal, Hospital Sant Joan de Déu i Clínic, Barcelona University, 08950 Barcelona, Spain; mizquierdo@sjdhospitalbarcelona.org (M.I.R.); cbalcells@sjdhospitalbarcelona.org (C.B.E.); bdelrey@sjdhospitalbarcelona.org (B.d.R.H.d.M.); 4 Institut de Recerca Sant Joan de Déu, 08950 Barcelona, Spain; clerin@fsjd.org (C.L.); mramonk@sjdhospitalbarcelona.org (M.R.-K.); 5 Endocrinology Department, Hospital Sant Joan de Déu, 08950 Barcelona, Spain

**Keywords:** growth failure, preterm infants, metabolic fingerprinting, multiplatform untargeted metabolomics

## Abstract

Very preterm infants (VPI, born at or before 32 weeks of gestation) are at risk of adverse health outcomes, from which they might be partially protected with appropriate postnatal nutrition and growth. Metabolic processes or biochemical markers associated to extrauterine growth restriction (EUGR) have not been identified. We applied untargeted metabolomics to plasma samples of VPI with adequate weight for gestational age at birth and with different growth trajectories (29 well-grown, 22 EUGR) at the time of hospital discharge. A multivariate analysis showed significantly higher levels of amino-acids in well-grown patients. Other metabolites were also identified as statistically significant in the comparison between groups. Relevant differences (with corrections for multiple comparison) were found in levels of glycerophospholipids, sphingolipids and other lipids. Levels of many of the biochemical species decreased progressively as the level of growth restriction increased in severity. In conclusion, an untargeted metabolomic approach uncovered previously unknown differences in the levels of a range of plasma metabolites between well grown and EUGR infants at the time of discharge. Our findings open speculation about pathways involved in growth failure in preterm infants and the long-term relevance of this metabolic differences, as well as helping in the definition of potential biomarkers.

## 1. Introduction

Prematurity is the leading cause of childhood morbidity and mortality. In very preterm infants (VPI), born at or before 32 weeks of gestation, postnatal growth failure is a frequent complication [1] that can worsen short [2] and long [3,4,5] term outcomes. Even though this condition can be improved through the optimization of feeding protocols [6,7,8] other non-nutritional known or unknown influences seem to contribute at least as much, as shown by the relatively small impact of nutritional variables in predicting growth impairment in VPI [1,9]. Furthermore, there is a lack of well-defined stand-alone markers for the prospective evaluation of nutrition and growth in the hospitalized preterm infant [10].

Metabolomic techniques aim to assess a whole array of small molecules present in a sample. This can be performed by selecting a subset of metabolites under a specific hypothesis (targeted metabolomics) or with a discovery (untargeted) approach, trying to identify most of the present components [11]. In recent years, these strategies have been used to search for biomarkers or to uncover underlying pathways in some adult [11], childhood [12], and even perinatal [13,14] conditions, with a special interest in metabolic diseases [15] and altered body composition [16,17].

Untargeted metabolomics relies on the application of a range of analytical technologies to simultaneously evaluate a broad spectrum of metabolites in biological matrices and it might be particularly suited for the neonatal population. It can cope with very small sample volumes, while producing a vast information which is necessary because metabolic pathways have been shown to differ from other stages of life [18], and targeted studies might miss the defining changes in these patients.

The methodology has already offered good insight into the development of biomarkers in stool in a cohort of VPI, by uncovering differences in the microbiome-associated fecal metabolome between healthy patients and those at risk of or suffering from late-onset sepsis (LOS) or necrotizing enterocolitis (NEC) [19,20]. Interestingly, the study of serum in the same individuals [21] did not identify discriminating compounds, suggesting that the success of these strategies requires careful selection of samples and a strict definition of the clinical condition under investigation. As a global process, growth would be expected to impact both serum and urinary profiles and, in fact, a few studies have indicated that this might be the case. Morniroli et al. [22], described some differences in the urine metabolome of VPI at term corrected age when compared to term infants and tried to correlate this to altered body composition. Younge et al. [23] applying mainly a targeted design, reported a longitudinal divergence in the profile of acylcarnitines between two groups of extremely preterm infants (EPI) according to their postnatal growth trajectory and suggesting that these may respond to altered gut microbiome development.

In this context, we aimed to apply a multiplatform untargeted metabolomic design with three complementary analytical platforms (liquid chromatography–mass spectrometry, gas chromatography–mass spectrometry and capillary electrophoresis–mass spectrometry) measuring compounds of different chemical nature in order to ensure maximum coverage for the investigation of metabolic signatures associated with growth restriction during hospital admission in a well-characterized cohort of very preterm infants fed predominantly breast milk. This could contribute to clarify relevant pathways and to identify compounds with potential for the development of future biomarkers.

## 2. Materials and Methods

### 2.1. Study Design 

Study of biochemical markers of postnatal growth restriction in a prospective cohort of very preterm newborns admitted within 24 hours of birth in a level III neonatal unit between February 2013 and April 2016. Exclusion criteria were death before discharge, major malformations, chromosomal abnormalities or genetic diseases and congenital infection. Only participants with no history of intrauterine growth restriction (IUGR) were selected for metabolomic analyses. The protocol was approved by the local ethics committee (PIC-95-13). Families of eligible babies were approached and signed a written informed consent for participation.

According to their postnatal growth trajectory until discharge, the infants were classified into normally grown or extrauterine growth restricted (EUGR: under the 10th centile or a z-score of −1.28 of weight-for-gestational age) according to local intrauterine growth curves [24] if under 40 weeks postmenstrual age (PMA) or the WHO standards [25] if over 40 weeks PMA. EUGR was further classified as moderate (between 3rd and 10th centile or z-score −1.88 and −1.28) or severe (under 3rd centile or z-score −1.88).

### 2.2. Sample Collection and Processing

On the days leading to discharge, and coinciding with collection for clinical reasons whenever possible, a volume of 0.5 mL of blood was drawn from a peripheral vein and collected in an EDTA tube. Efforts were taken to keep extraction times to early morning around 9 am and in a fasted state (2–3 h after the last feeding). The sample was immediately centrifuged (3000 rpm for 5 min) and plasma frozen at –80 °C until further processing.

### 2.3. Untargeted Metabolomics Analysis

#### 2.3.1. Chemicals

Organic solvents (MS grade), analytical grade formic acid 99%, standard mix for GC-MS containing grain fatty acid methyl ester (FAME) mixture (C8:0–C22:1n9) and chemical standards were from Sigma-Aldrich. Sialylation-grade pyridine was from VWR International BHD Prolabo (Madrid, Spain). Reference mass solutions for LC-MS and CE-MS were from Agilent Technologies. Ultrapure water (Milli-Qplus185 system Millipore, Billerica, MA, USA) was used in preparation of all buffers and standard solutions.

#### 2.3.2. Metabolite Extraction

Metabolite extraction was performed according to standard protocols [26,27,28]. Briefly, for LC-MS 50 µL of plasma was mixed with 350 µL of the solvents, methanol (175 µL) and MTBE (methyl *tert*-butyl ether) (175 µL) followed by centrifugation (4000 g, 15 °C, 15 min). For GC-MS analysis, proteins were precipitated by mixing 1 volume of plasma with 3 volumes of cold acetonitrile (1:3), followed by methoximation with O-methoxyamine hydrochloride (15 mg/mL) in pyridine, and silylation with N,O bis(trimethylsilyl)trifluoroacetamide (BSTFA) with 1% trimethylchlorosilane (TMCS). 10 ppm C18:0 methyl ester in heptane was used as internal standard. For CE-MS, 100 µL of plasma was mixed with 100 µL of 0.2 M formic acid that contained 5% acetonitrile and 0.4 mM methionine sulfone as internal standard. The sample was transferred to a centrifree ultracentrifugation device (Millipore Ireland Ltd., Carrigtohill, Ireland) with a 30 kDa protein cutoff for deproteinization through centrifugation (2000 g, 4 °C, 70 min).

Quality control (QC) samples were independently prepared for each technique by pooling equal volumes of each sample and following the same extraction procedure as applied for experimental samples. Analyte-free extraction blank samples were also prepared for each analytical run. All samples were randomized independently for metabolite extraction and for corresponding analytical run [29].

#### 2.3.3. Separation and Detection

In order to assess system suitability, blank samples were analyzed at the beginning/end of each run. Quality control samples were injected in order to condition each analytical platform, as well as to check metabolite profile and signal intensity. QC was rerun at the beginning/end, and after every six experimental samples to provide a measurement of the system’s stability, performance and reproducibility throughout the entire analysis [29].

#### 2.3.4. Untargeted Metabolomics by LC-TOF-MS

An UHPLC system (Agilent 1290 Infinity LC System, Waldbronn, Germany), equipped with a degasser, two binary pumps, and a thermostated autosampler coupled with Q-TOF LC/MS (6545) system (Agilent), was used in the ESI+ (positive electrospray ionization) and ESI− (negative electrospray ionization) mode to increase the number of detected metabolite ions. Briefly, 0.5 μL of sample was injected into a thermostated (60 °C) Agilent Poroshell 120 EC-C8 column (150 mm × 2.1 mm, 2.7 μm) with a guard column Ascentis® Express C8 (5 mm × 2.1 mm, 2.7). The flow rate was 0.5 mL/min with solvent A (10 mM ammonium formate in Milli-Q water), and solvent B (10 mM ammonium formate in methanol with 15% of isopropanol) for analysis in positive ionization mode, and solvent A (Milli-Q water with 0.1% formic acid), and solvent B (methanol with 0.1% formic acid and 15% of isopropanol) for analysis in negative ionization mode. Initial conditions at time 0 were 75% B, increasing to 96% B at 23 min. This condition was held until 31 min. The gradient then increased to 100% B by 31.5 min and held until 32.5 min. The system was returned to starting condition by 33 min, followed by a 7 min re-equilibration time, with a total run time of 40 min. Capillary voltage was set to 3.5 kV for positive and negative ionization mode; the drying gas flow rate was 11 L/min at 290 °C and gas nebulizer at 40 psi; fragmentor voltage was 175 V; skimmer and octopole radio frequency voltage (OCT RF Vpp) were set to 65 V and 750 V, respectively. Data were collected in the centroid mode at a scan rate of 1.0 scan/s. Mass spectrometry detection was performed in both positive and negative ESI mode in full scan from m/z 50 to 1000. The reference mass ions m/z 121.0509 (C5H4N4) and m/z 922.0098 (C18H18O6N3P3F24) in positive ionization mode or m/z 112.98568 (C2O2F3(NH4)) and m/z 1033.9881 (C18H18O6N3P3F24) in negative ionization mode were continuously infused by an automated Calibrant Delivery System (CDS), using a Dual Agilent Jet Stream Electrospray Ionization (Dual AJS ESI) source that continuously introduces a calibrant solution. The analytical conditions were applied according to the method developed by Villaseñor et al. with modifications [28].

#### 2.3.5. Untargeted Metabolomics by GC-Q-MS

A GC system (Agilent Technologies 7890A), equipped with an autosampler (Agilent 7693) and interfaced to an inert mass spectrometer with triple-Axis detector (5975C, Agilent), was used for analysis. Briefly, 2 μL of the derivatized sample was injected in a GC column DB5-MS (30 m length, 0.25 mm, 0.25 μm film 95% dimethyl/ 5% diphenylpolysiloxane) coupled to a pre-column (10 m J&W integrated with Agilent 122-5532G). The injector port was held at 250 °C, and the helium carrier gas flow rate was set at 1.0 mL/min. The split ratio was 1:10. The temperature gradient was programmed for an initial oven temperature of 60 °C (held for 1 min), increased to 325 °C at a rate of 10 °C/min; the system was allowed to cool down for 10 min before the next injection. The detector transfer line, the filament source and the quadrupole temperature were set to 280 °C, 230 °C and 150 °C, respectively. MS detection was performed in electron impact (EI) mode at −70 eV. The mass spectrometer was operated in scan mode over a mass range of m/z 50–600 at a rate of 2.7 scan/s [26].

#### 2.3.6. Untargeted Metabolomics by CE-TOF-MS 

An Agilent 7100 (CE) system, coupled to a TOF Mass Spectrometer (6224 Agilent) with electrospray ionization source, was used for sample analysis. A 1200 series ISO Pump from Agilent Technologies is used to supply sheath liquid. In brief, a fused-silica capillary (Agilent Technologies; total length, 96 cm; i.d., 50 μm) was pre-conditioned with 1 M NaOH for 30 min, followed by MilliQ® (Molsheim, France) water for 30 min and background electrolyte-BGE (1.0 M formic acid in 10% methanol) for 30 min. Before each analysis, the capillary was flushed for 5 min (950 mbar pressure) with BGE. Samples were injected at 50 mbar for 50 s. After each injection, along with the samples, BGE was co-injected for 20 s at 100 mbar pressure to improve reproducibility. Separations were performed at a pressure of 25 mbar and a voltage of 30 kV; current under these conditions was 20 μA. The MS was operated in positive mode, with a full scan from m/z 60 to 1000 at a rate of 1 scan/s. Drying gas was set to 10 L/min, nebulizer to 10 psi, voltage to 3.5 kV, fragmentor to 125 V, gas temperature to 200 °C and skimmer to 65 V. The sheath liquid composition was methanol/water (1/1, v/v), containing 1.0 Mmol/L formic acid with two reference masses (m/z 121.050873—purine (C5H4N4) and m/z 922.009798—HP-921 (C18H18O6N3P3F24)), which allows for mass correction and provides more accurate determination. Flow rate was 0.6 mL/min and split was set to 1/100 [27].

### 2.4. Data Management

#### 2.4.1. Metabolomics Data Processing

LC-MS and CE-MS data were cleaned from background noises and unrelated ions by recursive analysis in Mass Hunter Profinder (B.08.00, Agilent Technologies, Santa Clara, CA, USA) software. In the first step, Molecular Feature Extraction (MFE), the algorithm performs chromatographic deconvolution to find all features in the analyzed samples and align across all the selected sample files using mass and retention/migration time. In the second step, MFE results are used to perform recursive feature extraction, where the Find by Ion (FbI) algorithm uses the median mass, median retention/migration time, and composite spectrum calculated from the aligned features to improve reliability. Spectral deconvolution with Agilent Unknown Analysis software (Ver. B.08.00. Agilent Technologies, Santa Clara, CA, USA) was used to extract the data acquired by GC-MS analysis. Alignment of drift (by retention time and mass) and data filtering were performed with the Mass Profiler Professional ver. B.12.1 (Agilent Technologies, Santa Clara, CA, USA) software. Assignment of the target ion and the qualifiers, entire batch pre-processing and manual inspection of the acquired data including peak area and RT integration was performed with Agilent MassHunter Quantitative Analysis (Ver. B.08.00, Agilent Technologies, Santa Clara, CA, USA).

#### 2.4.2. Quality Assurance Procedure

Quality control and quality assurance procedures were applied according to published guidelines [29]. Acquired data were evaluated by examination of reproducibility of sample treatment procedure and analytical performance by raw data inspection. Principal component analysis (PCA-X), a projection method was used to check for signal drift, variation in QC samples and outliers. 

Tight clustering of QCs was observed for data acquired in all experiments, indicating high precision of the analytical outcome (Figure S1). Shewhart control charts were used to plot acquired signals versus the sample acquisition order to overview the analytical precision. Variation within measurements was calculated for QCs and expressed as relative standard deviation (RSD). Data was evaluated by Hotelling´s T2 Range Plot on PCA-X model and outlying observations were removed from further calculations.

### 2.5. Data Pre-Treatment

Data Filtration and Normalization

Before statistical analysis, filtration and data normalization were performed. Features with mean blank values higher than 10% of the mean value in samples were considered as non-relevant [29]. Variation of the compound concentrations in QC samples expressed as relative standard deviation (%RSD) was calculated and cut-off threshold of 20% for LC-MS and CE-MS and 30% for GC-MS was set for the RSD values of metabolites present in the QC samples. Instrumental variation detected in LC-MS data and CE-MS was corrected by QC samples applying support vector regression algorithm (QC-SVRC) [30]. GC-MS data was normalized according to the intensity of IS. IS normalization was also considered in order to correct for the unwanted variance related to sample preparation in CE-MS.

### 2.6. Statistical Analysis

Data normality was verified by Kolmogorow−Smirnov−Lillefors test and variance ratio by the Levene’s test. Clinical characteristics were summarized as number and percentage for categorical variables and differences between groups were assessed by chi-square tests with Fisher´s exact correction when appropriate. Continuous variables were represented by their mean and standard deviation and comparisons performed by Student´s t tests. All statistical analyses were undertaken with SPSS^®^ (IBM, New York, NY, USA) v25.

For metabolomics data, principal components analysis (PCA-X) and orthogonal projection to latent structures discriminant analysis (OPLS-DA) as well as other multivariate calculations and plots were performed in SIMCA-P + 14.0 (Umetrics, Umea, Sweden) in order to examine the data in multivariate settings. Combination of VIP-p(corr) (correlation coefficient combined with VIP, Variable Influence on the Projection) based on selected OPLS-DA model was applied for specified interpretations with the threshold for variable selection set to VIP > 1.0 and p(corr) > 0.5.

Metabolomic differences among experimental groups were tested by using either the ANOVA or the Kruskal−Wallis tests according to normality of the variable distribution, with post hoc test for multiple comparisons. The level of statistical significance was set at 95% (*p* < 0.05) and false discovery rate set at 0.05. Univariate statistical analyses were performed with GraphPadPrism^®^ (GraphPad Software Inc., San Diego, CA, USA) version 7.04.

MetaboAnalyst, a comprehensive web-based tool for metabolomic data analysis, visualization, and functional interpretation was used to test associations between variables and clinical metadata for hierarchical heat map clustering [31].

### 2.7. Metabolite Identification

For metabolite identity assignment, accurate m/z measurements of detected chromatographic peaks from LC-MS and CE-MS data were first matched to metabolites from online MS databases as Kegg, Metlin, LipidMaps, and HMDB using advanced CEU Mass Mediator tool [32]. Isotopic distributions for each metabolite feature (LC-MS and CE-MS) have been studied for the confirmation. For LC-MS data, AutoMSMS mode was used to obtain MS and MS/MS spectra of the three most abundant precursor ions per cycle. Final metabolite assignment according to fragmentation pattern was dependent on the ability to obtain mass spectra with adequate signal. An in-house developed CE-MS standards library was used to compare relative migration time of selected metabolites and compound identification was confirmed by using chemical standards, if available. For GC-MS data compound identification was performed with the target metabolite Fiehn GC-MS Metabolomics RTL (Retention Time Locked) library (G1676AA, Agilent), the CEMBIO-library and the NIST (National Institute of Standards and Technology) mass spectra library (Ver. 2014), using the ChemStation software and native PBM (Probability-Based Matching) algorithm (G1701EA E.02.00.493, Agilent Technologies, Santa Clara, CA, USA).

## 3. Results 

### 3.1. Clinical Characteristics of the Study Subjects

We selected plasma samples obtained from 51 VPIs previous to discharge. Twenty-nine corresponded to preterm babies with weight at discharge over the 10th percentile for postmenstrual age and 22 to EUGR patients.

There were no significant differences in gender or gestational age at birth. Although prevalence of complications of prematurity were in general higher in EUGR infants, none of these differences were significant (see Table S1). There was a trend for a higher postmenstrual age at discharge in the EUGR group (36.9 ± 2.0 vs 38.0 ± 2.1 weeks, *p* = 0.075).

Growth between the groups was different from birth. Weight and head circumference and their z-scores for both parameters at birth were lower in infants that went on to develop EUGR (weight: 1223 ± 235g vs 1402 ± 294g, *p* = 0.023 and z-scores –0.34 ± 0.74 vs 0.42±0.68, *p* = 0.027; head circumference (HC) 25.9 ± 2.0cm vs 26.9 ± 1.9 cm, p 0.084 and z-scores –0.55 ± 0.74 vs –0.09 ± 0.57, *p* = 0.023) and this difference kept increasing during admission, with bigger falls in z-score for both weight and head circumference from birth to discharge (fall in weight z-score –1.88 ± 0.61 in EUGR vs –1.25 ± 0.61 in normally grown, *p* = 0.001; fall in HC z-score –1.07 ± 1.19 in EUGR vs – 0.42 ± 0.67 in normally grown, *p* = 0.033).

Global nutrition during the 1st week of life was similar between groups, but supply was mainly intravenous in the EUGR group and enteral in the non-EUGR group (see Table 1). During the 2nd week of life energy and protein supply was lower in the EUGR group (109.0 ± 16.0 vs 120.6 ± 14.8 kcal/kg/day, *p* = 0.010 and 3.2 ± 0.8 vs 3.6 ± 0.7 g/kg/day, *p* = 0.069), mostly at the expense of enteral nutrition (see Table 1). There were no differences in type of feeding at discharge (69.0% of non-EUGR and 77.3% of EUGR were only receiving their own mother’s milk, *p* = 0.510).

### 3.2. Metabolic Fingerprinting

**Figure 1:**
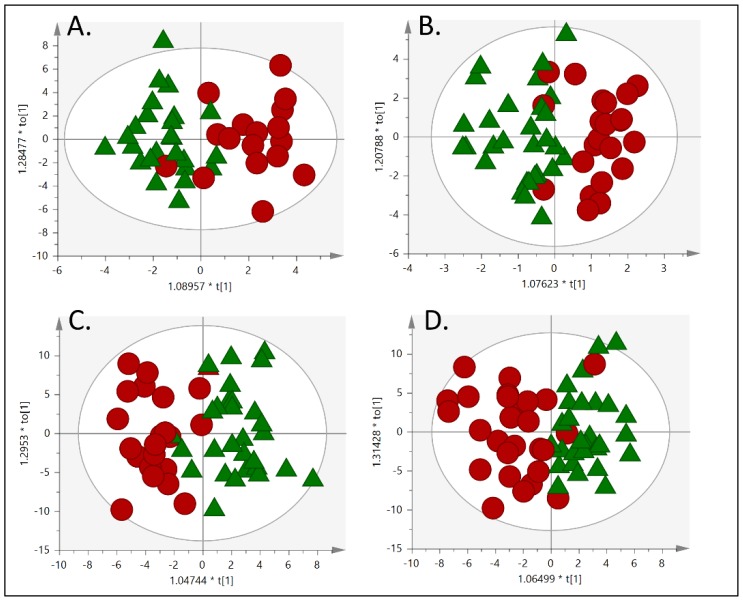
OPLS-DA scores plot (**A**) capillary electrophoresis-mass spectrometry, (R2 = 0.66, Q2 = 0.2, CV-ANOVA *p* = 0.026); (**B**) gas chromatography-mass spectrometry, (R2 = 0.63, Q2 = 0.034, CV- ANOVA *p* = 0.036); (**C**) liquid chromatography-mass spectrometry/ESI+,(R2 = 0.48, Q2 = -0.006, CV- ANOVA, *p* = 1.000); and (**D**) liquid chromatography-mass spectrometry /ESI- (R2 = 0.62, Q2 = −0.00006, CV- ANOVA *p* = 0.046). R2 = coefficient for variance explained; Q2 = coefficient for variance predicted. ▲ non-EUGR, ● EUGR.

Applying an untargeted strategy, a significant amount of information was obtained from the metabolomic study of plasma, resulting in 1040 and 1044 metabolic features from LC-MS operated in positive and negative ionization mode, respectively, 409 acquired from CE-MS and 96 from GC-MS analysis. This data matrix was used for further data treatment. Considering the highly dimensional structure of metabolomics data, with low sample-to-variable ratio, and many uninformative or redundant variables, strategies for dimensionality reduction were applied. Using orthogonal partial least squares discriminant analysis (OPLS-DA) fitted models we explored differences in metabolic phenotypes between non-EUGR and EUGR cases. All generated models (Figure 1) present good separation between groups (R2), but rather poor predictivity (Q2). However, despite this fact, the models established for CE-MS, GC-MS and LC-MS/ESI- presented significant values of CV-ANOVA, which is considered as a measure of significance for the observed group separation. Moreover, as for the purpose of this global and exploratory analysis and considering that only the first component holds group separation, we examined which variables differentiated between groups and could be evaluated in subsequent univariate analysis.

**Figure 2:**
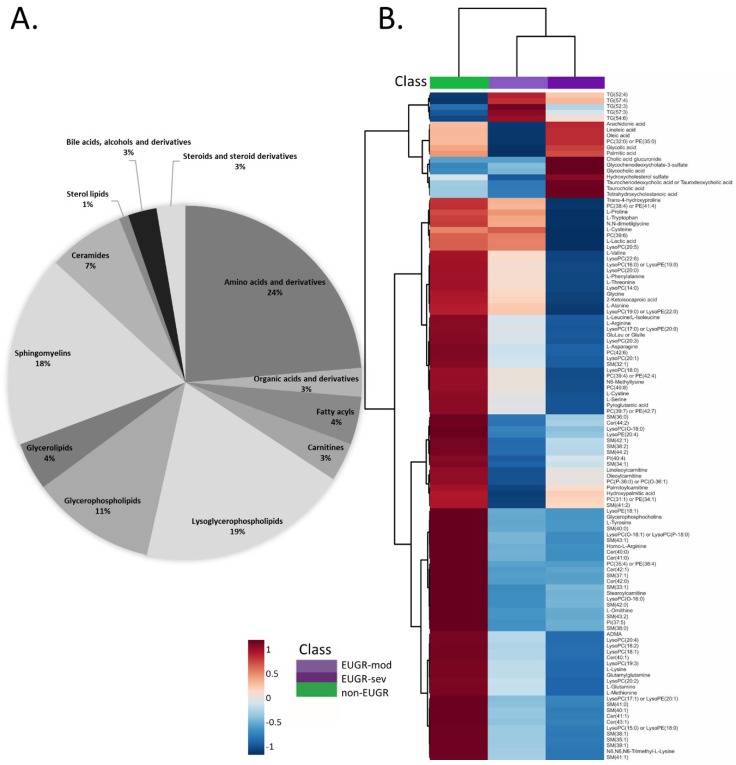
(**A**) Panel A. Pie chart representing the distribution of metabolites identified from multiplatform metabolomic analysis that were significantly different between EUGR and non EUGR individuals. (**B**) Panel B. A hierarchical clustering with heatmap using Euclidean distance measure and Ward clustering algorithm has been applied for statistically significant metabolites, illustrating the differences in the metabolite abundance between non-EUGR, EUGR-mod and EUGR-sev cases. Each colored cell corresponds to an abundance value, where blue indicates the lowest and red the highest value.

Variable influence on the projection (VIP) scores as a quantitative estimation of the discriminatory power of each individual metabolite were extracted only for the predictive component of the OPLS-DA model. Additionally, a complementary p(corr) correlation coefficient combined with VIP (VIP-p(corr) analysis) allowed the selection of the most relevant metabolites for the separation. For further evaluation selected metabolic features were subtracted for univariate, either the ANOVA or the Kruskal−Wallis with post hoc Benjamini-Hochberg (FDR, false discovery rate) correction for multiple comparisons, resulting in 114 compounds found to be statistically significant. The pie chart presented (Figure 2A) shows the percentage distribution of the specified metabolite classes illustrating the diversity of metabolites that were found to be associated with EUGR. Of all reported compounds, 24% were amino acids and derivatives detected in CE-MS and/or GC-MS and 30% were glycerol- and lysoglycero-phospholipids (including phosphatidylinositols, lysophosphatidylcholines, and lysophosphatidylethanolamines) detected in LC-MS positive and/or negative mode. Additionally, sphingolipids, including ceramides (7%) and sphingomyelins (18%) with different backbone lengths and degree of unsaturation were identified by LC-MS. Free fatty acids and hydroxyl fatty acid (hydroxypalmitic acid) were another lipid class detected in negative LC-MS mode and differentially present between groups. Fatty acids, namely oleic acid, linoleic acid and palmitic acid were also detected in GC-MS and LC-MS negative mode. Long-chain acyl fatty acid derivative esters of carnitine (linoleylcarnitine, palmitoylcarnitine, oleoylcarnitine, stearoylcarnitine) were detected in positive LC-MS mode.

A heatmap with hierarchical clustering was constructed to visualize the differences in the average intensities of statistically significant metabolites (Figure 2B). A clear metabolic pattern discriminating between non-EUGR and EUGR cases can be observed, with a gradient according to the degree of EUGR severity (EUGR-mod and EUGR-sev) for many compounds. A heatmap analysis indicates that most of the reported metabolites were downregulated in EUGR cases. However, in the case of steroids and steroid derivatives or fatty acids we could observe an increase in the associated relative signal intensities in samples from EUGR babies.

Metscape, a bioinformatics framework for the exploration of experimental metabolomics and expression profiling data in the context of human metabolism [33] was used to identify and visualize enriched pathways from acquired metabolomics data (Figure S2). This allows a deeper insight into the molecular pathways that might be related to EUGR in preterm infants. The main players were related to amino acid metabolism and mapped as (1) urea cycle and metabolism of arginine, proline, glutamate, aspartate and asparagine; (2) tyrosine metabolism; (3) tryptophan metabolism; (4) valine, leucine and isoleucine metabolism; (5) lysine metabolism; (6) methionine and cysteine metabolism; and (7) glycine, serine, alanine and threonine metabolism. Additionally, our data point to alterations in lipid molecular pathways, particularly glycerophospholipid and glycosphingolipid metabolism. Other pathways involved were those associated with bile acid biosynthesis, fatty acid metabolism, purine metabolism or glycolysis and gluconeogenesis.

**Figure 3:**
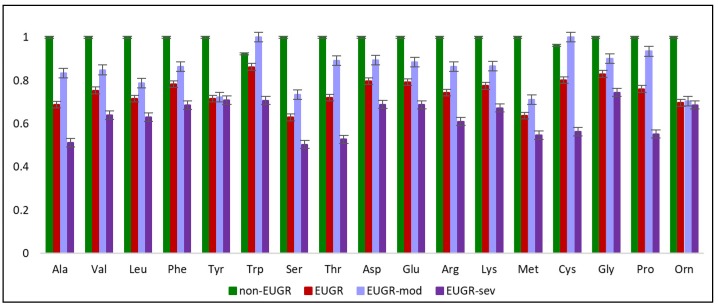
The scaled relative abundance of statistically significant amino acids reflecting the differences between non-EUGR and EUGR, together with stratification for moderate and severe EUGR cases.

A detailed inter-group comparison of the compounds belonging to the classes of amino acids and derivatives and lipid-related molecules is presented in Tables S2,S3, respectively. All essential and non-essential amino acids, except for histidine, aspartic acid and glutamic acid were significantly different between EUGR and non-EUGR patients, and most showed a progressive decrease along with the degree of growth restriction (Figure 3).

Interestingly, some biochemical changes seemed to be specifically present in infants with the most severe degree of growth failure, mainly regarding the levels of steroids and steroid derivatives and bile acids, particularly taurocholic acid (Table S3).

## 4. Discussion

### 4.1. EUGR is Associated With Disrupted Amino Acid Metabolism

The plasmatic levels of most essential and non-essential amino acids (leucine/isoleucine, lysine, methionine, phenylalanine, threonine, valine, arginine, alanine, tyrosine, glutamine, glycine, proline, serine, asparagine) were higher in well-grown VPI, and there was a gradient according to the severity of EUGR (moderate > severe).

An increase in plasma branched-chain amino acids (BCAA) has been described in relation to obesity and insulin resistance in both adults [34] and prepubertal children [15]. In preterm infants, better in-hospital growth should result in a higher mass of insulin-sensitive tissue (muscle and adipose tissue), and in principle, lower peripheral resistance [35]. Actually, it has been shown that a higher provision of amino acids can induce insulin secretion in VPIs [35] and this does in turn improve levels of insulin-like growth factor I [36] (IGF-I). IGF-I is higher in healthy adults with lower body size than in obese insulin-resistant individuals [34], but it correlates with both fetal and postnatal mass [37], so it makes more sense that its associated metabolic markers will represent a more adequate pattern of growth in the perinatal context.

Additionally, BCAA, particularly leucine, can be biomarkers of reduced muscle mass and performance [38,39] in the elderly adult. When compared with healthy newborns, preterm infants show a deficit in lean mass by the time they reach term-equivalent age [40,41]. The studies in elderly individuals suggest that lower BCAAs might reflect differences in global protein consumption, arguing against correction of intake by body weight in cases of low muscle mass [38] and proposing that an absolute minimum intake, related to the plasma and intramuscular levels of leucine, is required to activate protein synthesis through the mTOR pathway [38]. Total protein intake was indeed lower in the EUGR groups in our sample (see Table 1).

Most reports of amino acid levels in prematurity in relation to growth describe global changes affecting them all in the same direction. In a randomized control trial (RCT) of a nutritional intervention, higher levels of plasma amino acids in very low birth weight infants at 5 weeks of life were related to higher growth velocity within the intermediate rate of protein supply (2.9–3.4 g/kg/day) [42]. This was specially true for BCAA and in the healthier patients (appropriate for gestational age at birth and without LOS or bronchopulmonary displasia (BPD) [42]. In the same study, preterm infants with LOS or BPD had higher levels of total amino acids, higher arginine levels and higher levels of aspartic acid [42]. In our study, respiratory morbidity was more prevalent in the growth restricted group (not statistically significant due to sample size), while the incidence of late onset sepsis LOS was the same, but amino acid levels were lower. These differences might be justified by the high prevalence of SGA in the RCT, while they were excluded in our study, or by the effects of the nutritional intervention. Contrary to our results, an inverse relationship between plasma amino acids and growth velocity in formula fed infants of higher gestational age were reported in an older investigation [43]. They speculate that this could be due to amino acid utilization during tissue accretion [43]. We tried to assess if our EUGR population was starting to experience some degree of catch up at the moment of discharge, but the data do not support this hypothesis. In their population, higher nitrogen retention was associated with higher plasma phenylalanine [43] (PHE). PHE was also higher in our well-grown preterms. A metabolomic investigation of EUGR in extreme prematures more specifically centered in a targeted analysis of carnitines found higher concentrations of glutamine/glutamic acid and proline in infants with growth failure and higher concentrations of methionine and histidine in infants with appropriate growth [23]. The concomitant analysis of the intestinal microbiome uncovered correlations between the amino acid profile and specific taxa [23]. The differences with our results might reflect differences in microbiota, in the gestational age of the samples (their study was performed in extremely premature infants, born under 28 weeks) or in the degree of growth restriction (the average weight percentile of our non-EUGR group was P20 and P3 for the EUGR, compared to P10 and <1, respectively, in their paper [23]).

### 4.2. EUGR is Associated With Disrupted Lipid Metabolism

EUGR VPI had lower plasma levels of several phospholipids (glycerophospholipids and sphingolipids), and for many of these the magnitude of the decrease corresponded with the severity of the growth failure. 

Increased levels of certain sphingolipids (sphingomyelins and ceramides) have been related to obesity and insulin resistance in adults [34], and a lifestyle intervention in children with obesity showed a reduction in sphingolipid levels together with, but not mediated by, beneficial effects on BMI [17]. Although the metabolomic profile of the normally grown preterm infants resembles that of older patients with overweight or insulin resistance, interpretation must be cautious as they are in a completely different developmental setting. Patients classified as “non-EUGR” had not had an excessive weight gain: at discharge, they were still below what would be expected for their PMA and their z-scores for weight and length had both decreased from birth (−1.25 ± 0.61 and −0.97 ± 0.90, respectively). Not much is known about lipid metabolism during infancy. The scarce information seems to highlight relevant differences with the adult age. Healthy breast-fed infants present profiles (high cholesterol, high LDL, low HDL) that would be considered adverse in adults [44]. Plasma lipids in infants contain a higher proportion of sphingomyelins [45], which are the predominant phospholipid in breast milk [46]. Sphingolipids are particularly relevant in brain development and function [47,48], and their lower availability might mediate the worsening of neurodevelopmental outcome after EUGR [49].

The lipid profile experiences rapid changes within the first 12 months of life [45], specifically characterized by an increase in lysophosphatidylcholines. LysoPCs were higher in the non-EUGR group and lowest in the severe EUGR, with no sizeable difference in their chronological age. This could then indicate that postnatal growth failure is associated to a delay in metabolic development, as previously proposed [23]. It has been postulated [44] that higher levels of proinflammatory lysoPCs in breastfed infants might play a role in protection from infectious disease. A history of LOS is frequently more prevalent in EUGR preterm infants [1], although this was not the case in our sample, maybe due to small sample size.

Interestingly, our study replicates the observation that levels of lysoPC (14:0) in the first months of life are associated with (and predictive of) faster growth [16]. This could be interpreted in the context of the rapid fat mass acquisition after preterm birth [41], which might be less pronounced in EUGR babies, and that normalizes to levels of term-born infants by 5 months corrected age [50]. On the contrary, we could not confirm other lipid species that have been proposed as markers of growth in term infants [44], maybe because they were described in the context of the different growth rates of breast- or formula-feeding and feeding type was homogeneous between groups in our sample.

In agreement with previous reports [23], some long-chain acylcarnitines were higher in the normally grown VPIs. Although in the adult this has been associated to insulin resistance [51], in the context of prematurity it may indicate a persistent state of malnutrition and delayed metabolic maturation that may relate to aberrant gut colonization [23]. At the time of sampling, both groups were receiving equivalent nutrition (Table 1), but this might have been insufficient to compensate for earlier lower protein and energy supply, or this early deficit might have had a “programming” effect on metabolism.

Several bile acid metabolites were increased in plasma of EUGR VPIs, specifically in the severe cases. Bile acids have been proposed as markers of liver injury [52]. Liver injury in preterm infants is multifactorial [53], but exposure of both groups to predisposing factors (infection, prolonged parenteral nutrition, prolonged fasting) was similar. It might be that infants suffering from postnatal growth restriction had a certain degree of subclinical hepatic dysfunction and contributing somehow to altered metabolism and growth, but our data are not enough to hypothesize further. Also, bile acid metabolism is closely related to the function of gut bacteria [54] and these disparity might reflect differences in intestinal colonization [23].

## 5. Conclusions

In summary, through a multiplatform untargeted metabolomic approach we have identified a range of metabolites associated to postnatal growth faltering in very preterm infants. The most relevant markers relate to amino acid metabolism, particularly regarding BCAA, but there are also striking global changes in lipid species.

Many of the alterations described in the preterm infants with better growth have been related to an adverse metabolic profile when studying adult or obese populations. In the context of our study, we believe that they represent a healthy metabolic profile associated to a physiologic period of rapid growth and fat deposition, and that they correspond to the comparison with a population presenting malnutrition and failure to thrive. Nevertheless, it will be interesting to analyze follow up data and see if this uncovers indeed a higher risk of insulin resistance and adverse cardiovascular outcome in the preterm infants with a better weight for PMA at discharge.

## Figures and Tables

**Table 1:** Comparison of nutritional and growth parameters between normally grown and extrauterine growth restriction (EUGR) patients. Categorical values are represented as number (%) and continuous variables as mean (SD). Categorical values were compared with chi-square tests and continuous variables with Student´s *t* tests.

		Normally Grown (*n* = 29)	EUGR (*n* = 22)	*p*-Value
At birth	Gestational age (weeks)	29.8 (1.8)	29.4 (1.9)	0.390
	Birthweight (g) Z-score	1402 (294) 0.42 (0.68)	1223 (235) –0.34 (0.74)	***0.023*** ***0.027***
	Length at birth (cm) Z-score	39.2 (3.0) 0.20 (0.80)	37.9 (2.6) –0.17 (0.76)	0.091 0.109
	Head circumference at birth (cm) Z-score	26.9 (1.9) –0.09 (0.57)	25.9 (2.0) –0.55 (0.74)	0.084 ***0.023***
At discharge	Postmenstrual age (weeks)	36.8 (2.1)	37.8 (2.2)	0.094
	Weight at discharge (g) Z-score Fall in weight z-score from birth	2399 (353) –0.83 (0.34) –1.25 (0.61)	2216 (444) –1.91 (0.43) –1.88 (0.61)	0.108 ***<0.0001*** ***0.001***
	Length at discharge (cm) Z-score Fall in length z-score from birth	45.5 (1.9) –0.78 (0.69) –0.97 (0.90)	45.3 (2.5) –38 (0.95) –1.14 (0.96)	0.796 ***0.012*** 0.535
	Head circumference at discharge (cm) Z-score Fall in HC z-score from birth	32.2 (1.6) –0.52 (0.58) –0.42 (0.67)	32.0 (1.4) –1.66 (1.08) –1.07 (1.19)	0.424 ***<0.0001*** ***0.033***
Nutrition	Parenteral nutrition (days)	10.1 (6.9)	12.3 (7.8)	0.293
	Age at first full enteral feeds (days)	10.6 (6.7)	11.8 (4.4)	0.461
	Average parenteral nutrition 1st week Energy (kcal/kg/day) Protein (g/kg/day) Lipids (g/kg/day) Protein/energy ratio (g/100kcal)	61.3 (12.2) 2.4 (0.7) 1.7 (0.7) 3.5 (0.5)	71.1 (11.1) 2.8 (0.5) 2.1 (0.5) 3.6 (0.3)	***0.014*** ***0.030*** ***0.018*** 0.100
	Average enteral nutrition 1st week Volume (ml/kg/day) Calculated energy (kcal/kg/day) Calculated protein (g/kg/day)	33.3 (21.2) 24.3 (16.0) 0.6 (0.4)	21.3 (14.4) 15.2 (10.2) 0.3 (0.2)	***0.021*** ***0.017*** ***0.019***
	Global nutrition 1st week (PN + enteral) Energy (kcal/kg/day) Protein (g/kg/day)	85.6 (8.9) 3.0 (0.5)	86.3 (8.5) 3.2 (0.3)	0.776 0.163
	Average parenteral nutrition 2nd week Energy (kcal/kg/day) Protein (g/kg/day) Lipids (g/kg/day) Protein/energy ratio (g/100kcal)	20.3 (30.4) 0.8 (1.2) 0.5 (0.9) 2.9 (0.5)	31.4 (30.0) 1.1 (1.1) 0.7 (0.9) 2.9 (0.5)	0.203 0.445 0.326 0.678
	Average enteral nutrition 2nd week Volume (mL/kg/day) Calculated energy (kcal/kg/day) Calculated protein (g/kg/day)	123.0 (47.4) 100.2 (41.6) 2.7 (1.3)	97.9 (42.7) 77.6 (37.1) 2.1 (1.2)	***0.056*** ***0.050*** ***0.083***
	Global nutrition 2nd week (PN + enteral) Energy (kcal/kg/day) Protein (g/kg/day)	120.6 (14.8) 3.6 (0.7)	109.0 (16.0) 3.2 (0.8)	***0.010*** ***0.069***
	Feeding at discharge Own Mother´s Milk Mixed feeding Formula	20 (69.0) 4 (13.8) 5 (17.2)	17 (77.3) 5 (22.7) 0 (0.0)	– * – –
	Exclusive own´s mother milk at discharge	20 (69.0)	17 (77.3)	0.510
	Nutritional intake at discharge Milk volume (ml/kg/day) Calculated protein intake Per kilogram (g/kg/day) Total (g/day) Calculated energy intake (kcal/kg/day)	170 (16) 2.3 (1.0) 5.5 (2.2) 127 (18)	163 (23) 1.9 (0.9) 4.3 (2.0) 124 (17)	0.222 0.143 0.049 0.520

* Chi-square *p* not calculated due to 50% of cells with an expected count of less than 5. Comparisons with a *p*–value < 0.05 were considered significant and are highlighted in bold and italic.

## Supplementary Materials

The following are available online at https://www.mdpi.com/2072-6643/12/4/1188/s1, Figure S1: PCA-X model for the prediction of pooled QC samples. Figure S2: Visual representation of metabolomics data consisting the network of the metabolites involved in EUGR process based on KEGG IDs reported for statistically significant compounds. Table S1: Comparison of clinical parameters between the normally grown and EUGR patients. Categorical values are represented as number (%) and continuous variables as mean (SD). Table S2. Differences in plasma amino acids and derivatives between well-grown and EUGR VPI. Table S3. Differences in plasma lipid species between well-grown and EUGR VPI.

## References

[1] Izquierdo Renau M., Aldecoa-bilbao V., Balcells Esponera C., del Rey Hurtado de Mendoza B., Iriondo Sanz M., Iglesias Platas I. (2019). Applying Methods for Postnatal Growth Assessment in the Clinical Setting: Evaluation in a Longitudinal Cohort of Very Preterm Infants. Nutrients.

[2] Ehrenkranz R.A., Das A., Wrage L.A., Poindexter B.B., Higgins R.D., Stoll B.J., Oh W. (2011). Early nutrition mediates the influence of severity of illness on extremely LBW infants. Pediatr. Res..

[3] Ehrenkranz R.A. (2006). Growth in the Neonatal Intensive Care Unit Influences Neurodevelopmental and Growth Outcomes of Extremely Low Birth Weight Infants. Pediatrics.

[4] Neubauer V., Griesmaier E., Pehböck-Walser N., Pupp-Peglow U., Kiechl-Kohlendorfer U. (2013). Poor postnatal head growth in very preterm infants is associated with impaired neurodevelopment outcome. Acta Paediatr. Int. J. Paediatr..

[5] Schneider J., Fumeaux C.J.F., Duerden E.G., Guo T., Foong J., Graz M.B., Hagmann P., Chakravarty M.M., Hüppi P.S., Beauport L. (2018). Nutrient intake in the first two weeks of life and brain growth in preterm neonates. Pediatrics.

[6] Cormack B.E., Bloomfield F.H. (2013). Increased protein intake decreases postnatal growth faltering in ELBW babies. Arch. Dis. Child. Fetal Neonatal Ed..

[7] Andrews E.T., Ashton J.J., Pearson F., Beattie R.M., Johnson M.J. (2019). Early postnatal growth failure in preterm infants is not inevitable. Arch Dis Child Fetal Neonatal Ed.

[8] Izquierdo M., Martínez-Monseny A.F., Pociello N., Gonzalez P., Del Rio R., Iriondo M., Iglesias-Platas I. (2016). Changes in Parenteral Nutrition during the First Week of Life Influence Early but Not Late Postnatal Growth in Very Low-Birth-Weight Infants. Nutr. Clin. Pract..

[9] Stevens T.P., Shields E., Campbell D., Combs A., Horgan M., La Gamma E.F., Xiong K.N., Kacica M. (2015). Variation in Enteral Feeding Practices and Growth Outcomes among Very Premature Infants: A Report from the New York State Perinatal Quality Collaborative. Am. J. Perinatol..

[10] Pereira-da-silva L., Virella D., Fusch C. (2019). Nutritional Assessment in Preterm Infants: A Practical Approach in the NICU. Nutrients.

[11] Wishart D.S. (2019). Metabolomics for investigating physiological and pathophysiological processes. Physiol. Rev..

[12] Khusial R.D., Cioffi C.E., Caltharp S.A., Krasinskas A.M., Alazraki A., Knight-Scott J., Cleeton R., Castillo-Leon E., Jones D.P., Pierpont B. (2019). Development of a Plasma Screening Panel for Pediatric Nonalcoholic Fatty Liver Disease Using Metabolomics. Hepatol. Commun..

[13] Dudzik D., Zorawski M., Skotnicki M., Zarzycki W., García A., Angulo S., Lorenzo M.P., Barbas C., Ramos M.P. (2017). GC–MS based Gestational Diabetes Mellitus longitudinal study: Identification of 2-and 3-hydroxybutyrate as potential prognostic biomarkers. J. Pharm. Biomed. Anal..

[14] Dudzik D., Revello R., Barbas C., Bartha J.L. (2015). LC - MS-based metabolomics identification of novel biomarkers of chorioamnionitis and its associated perinatal neurological damage. J. Proteome Res..

[15] Mastrangelo A., Martos-Moreno G., García A., Barrios V., Rupérez F.J., Chowen J.A., Barbas C., Argente J. (2016). Insulin resistance in prepubertal obese children correlates with sex-dependent early onset metabolomic alterations. Int. J. Obes..

[16] Rzehak P., Hellmuth C., Uhl O., Kirchberg F.F., Peissner W., Harder U., Grote V., Weber M., Xhonneux A., Langhendries J.P. (2014). Rapid growth and childhood obesity are strongly associated with LysoPC(14:0). Ann. Nutr. Metab..

[17] Leal-Witt M.J., Ramon-Krauel M., Samino S., Llobet M., Cuadras D., Jimenez-Chillaron J.C., Yanes O., Lerin C. (2018). Untargeted metabolomics identifies a plasma sphingolipid-related signature associated with lifestyle intervention in prepubertal children with obesity. Int. J. Obes..

[18] Vlaardingerbroek H., Roelants J.A., Rook D., Dorst K., Schierbeek H., Vermes A., Vermeulen M.J., van Goudoever J.B., van den Akker C.H.P. (2014). Adaptive regulation of amino acid metabolism on early parenteral lipid and high-dose amino acid administration in VLBW infants - A randomized, controlled trial. Clin. Nutr..

[19] Stewart C.J., Embleton N.D., Marrs E.C.L., Smith D.P., Fofanova T., Nelson A., Skeath T., Perry J.D., Petrosino J.F., Berrington J.E. (2017). Longitudinal development of the gut microbiome and metabolome in preterm neonates with late onset sepsis and healthy controls. Microbiome.

[20] Stewart C.J., Embleton N.D., Marrs E.C.L., Smith D.P., Nelson A., Abdulkadir B., Skeath T., Petrosino J.F., Perry J.D., Berrington J.E. (2016). Temporal bacterial and metabolic development of the preterm gut reveals specific signatures in health and disease. Microbiome.

[21] Stewart C.J., Nelson A., Treumann A., Skeath T., Cummings S.P., Embleton N.D., Berrington J.E. (2016). Metabolomic and proteomic analysis of serum from preterm infants with necrotising entercolitis and late-onset sepsis. Pediatr. Res..

[22] Morniroli D., Dessì A., Giannì M.L., Roggero P., Noto A., Atzori L., Lussu M., Fanos V., Mosca F. (2019). Is the body composition development in premature infants associated with a distinctive nuclear magnetic resonance metabolomic profiling of urine?. J. Matern. Neonatal Med..

[23] Younge N.E., Newgard C.B., Cotten C.M., Goldberg R.N., Muehlbauer M.J., Bain J.R., Stevens R.D., O’Connell T.M., Rawls J.F., Seed P.C. (2019). Disrupted Maturation of the Microbiota and Metabolome among Extremely Preterm Infants with Postnatal Growth Failure. Sci. Rep..

[24] Programa de Salut Maternoinfantil, Direcció General de Salut Pública, D. de S (2008). Corbes de referència de pes, perímetre cranial i longitud en néixer de nounats d ’ embarassos únics, de bessons i de trigèmins a Catalunya.

[25] de Onis M., Martorell R., Garza C., Lartey A., Members of the WHO Multicentre Growth Reference Study Group (2006). WHO Child Growth Standards based on length/height, weight and age. Acta Paediatr..

[26] Garcia A., Barbas C., Metz T. (2011). Gas Chromatography-Mass Spectrometry (GC-MS)-Based Metabolomics. Metabolic Profiling. Methods in Molecular Biology.

[27] Naz S., Garcia A., Rusak M., Barbas C. (2013). Method development and validation for rat serum fingerprinting with CE-MS: Application to ventilator-induced-lung-injury study. Anal. Bioanal. Chem..

[28] Villaseñor A., Garcia-perez I., García A., Posma J.M., Andreas N.J., Modi N., Holmes E., Barbas C. (2014). phase extrac-tion, multiplatform analytical approach Breast milk metabolome characterization in a single phase extrac- tion, multiplatform analytical approach. Anal Chem..

[29] Dudzik D., Barbas-Bernardos C., García A., Barbas C. (2018). Quality assurance procedures for mass spectrometry untargeted metabolomics. a review. J. Pharm. Biomed. Anal..

[30] Kuligowski J., Sánchez-Illana Á., Sanjuán-Herráez D., Vento M., Quintás G. (2015). Intra-batch effect correction in liquid chromatography-mass spectrometry using quality control samples and support vector regression (QC-SVRC). Analyst.

[31] Chong J., Soufan O., Li C., Caraus I., Li S., Bourque G., Wishart D.S., Xia J. (2018). MetaboAnalyst 4.0: Towards more transparent and integrative metabolomics analysis. Nucleic Acids Res..

[32] Gil-de-la-Fuente A., Godzien J., Saugar S., Garcia-Carmona R., Badran H., Wishart D.S., Barbas C., Otero A. (2019). CEU Mass Mediator 3.0: A Metabolite Annotation Tool. J. Proteome Res..

[33] Karnovsky A., Weymouth T., Hull T., Glenn Tarcea V., Scardoni G., Laudanna C., Sartor M.A., Stringer K.A., Jagadish H.V., Burant C. (2012). Metscape 2 bioinformatics tool for the analysis and visualization of metabolomics and gene expression data. Bioinformatics.

[34] Newgard C.B., An J., Bain J.R., Muehlbauer M.J., Stevens R.D., Lien L.F., Haqq A.M., Shah S.H., Arlotto M., Slentz C.A. (2009). A BCAA Related Metabolic Signature that differentiates obese and lean humans contributes to insulin resistance. Cell Metab..

[35] Morgan C. (2015). The potential risks and benefits of insulin treatment in hyperglycaemic preterm neonates. Early Hum. Dev..

[36] Beardsall K., Vanhaesebrouck S., Frystyk J., Ogilvy-Stuart A.L., Vanhole C., van Weissenbruch M., Midgley P., Thio M., Cornette L., Gill B. (2014). Relationship between insulin-like growth factor I levels, early insulin treatment, and clinical outcomes of very low birth weight infants. J. Pediatr..

[37] Hellström A., Ley D., Hansen-Pupp I., Hallberg B., Ramenghi L.A., Löfqvist C., Smith L.E.H., Hård A.L. (2016). Role of Insulinlike Growth Factor 1 in Fetal Development and in the Early Postnatal Life of Premature Infants. Am. J. Perinatol..

[38] Ottestad I., Ulven S.M., Oyri L.K.L., Sandvei K.S., Gjevestad G.O., Bye A., Sheikh N.A., Biong A.S., Andersen L.F., Holven K.B. (2018). Reduced plasma concentration of branched-chain amino acids in sarcopenic older subjects: A cross-sectional study. Br. J. Nutr..

[39] Yamada M., Kimura Y., Ishiyama D., Nishio N., Tanaka T., Ohji S., Otobe Y., Koyama S., Sato A., Suzuki M. (2018). Plasma Amino Acid Concentrations Are Associated with Muscle Function in Older Japanese Women. J. Nutr. Heal. Aging.

[40] Johnson M.J., Wootton S.A., Leaf A.A., Jackson A.A. (2012). Preterm Birth and Body Composition at Term Equivalent Age: A Systematic Review and Meta-analysis. Pediatrics.

[41] Al-Theyab N.A., Donovan T.J., Eiby Y.A., Colditz P.B., Lingwood B.E. (2019). Fat trajectory after birth in very preterm infants mimics healthy term infants. Pediatr. Obes..

[42] Strømmen K., Haag A., Moltu S.J., Veierød M.B., Blakstad E.W., Nakstad B., Almaas A.N., Brække K., Rønnestad A.E., Daniel H. (2017). Enhanced nutrient supply to very low birth weight infants is associated with higher blood amino acid concentrations and improved growth. Clin. Nutr. ESPEN.

[43] Scott P.H., Berger H.M., Wharton B.A. (1985). Growth velocity and plasma amino acids in the newborn. Pediatr. Res..

[44] Prentice P., Koulman A., Matthews L., Acerini C.L., Ong K.K., Dunger D.B. (2015). Lipidomic analyses, breast- and formula-feeding, and growth in infants. J. Pediatr..

[45] Koulman A., Prentice P., Wong M.C.Y., Matthews L., Bond N.J., Eiden M., Griffin J.L., Dunger D.B. (2014). The development and validation of a fast and robust dried blood spot based lipid profiling method to study infant metabolism. Metabolomics.

[46] Cilla A., Diego Quintaes K., Barberá R., Alegría A. (2016). Phospholipids in Human Milk and Infant Formulas: Benefits and Needs for Correct Infant Nutrition. Crit. Rev. Food Sci. Nutr..

[47] Olsen A.S.B., Færgeman N.J. (2017). Sphingolipids: Membrane microdomains in brain development, function and neurological diseases. Open Biol..

[48] Liu H., Radlowski E.C., Conrad M.S., Dilger R.N., Johnson R.W. (2014). Early Supplementation of Phospholipids and Gangliosides Affects Brain and Cognitive Development in Neonatal Piglets Differences in cognitive development: Breast fed ( BF ) vs Formula fed ( FF ) BF children show higher cognitive development scores compa. J. Nutr..

[49] Belfort M., Gillman M., Buka S., McCormick M. (2013). Preterm infant linear growth and adiposity gain: Tradeoffs for later weight status, and IQ. J. Pediatr..

[50] Roggero P., Giannì M.L., Amato O., Orsi A., Piemontese P., Cosma B., Morlacchi L., Mosca F. (2008). Postnatal growth failure in preterm infants: Recovery of growth and body composition after term. Early Hum. Dev..

[51] McCoin C.S., Knotts T.A., Adams S.H. (2015). Acylcarnitines-old actors auditioning for new roles in metabolic physiology. Nat. Rev. Endocrinol..

[52] Luo L., Aubrecht J., Li D., Warner R.L., Johnson K.J., Kenny J., Colangelo J.L. (2018). Assessment of serum bile acid profiles as biomarkers of liver injury and liver disease in humans. PLoS ONE.

[53] Satrom K., Gourley G. (2016). Cholestasis in Preterm Infants. Clin. Perinatol..

[54] Ridlon J.M., Kang D.J., Hylemon P.B., Bajaj J.S. (2014). Bile acids and the gut microbiome. Curr. Opin. Gastroenterol..

