# Supplementary material for: Plasma Metabolome Alterations Associated with Extrauterine Growth Restriction
Source: Nutrients. 2020 Apr 23;12(4):1188. doi: 10.3390/nu12041188 (PMC7230608; doi:10.3390/nu12041188)
Supplement: Supplementary file 1 [file nutrients-12-01188-s001.pdf]

Table S1. Comparison of clinical parameters between the normally grown and EUGR patients. Categorical values are represented as number (%) and continuous variables as mean (SD). Significance was assessed with *Chi-Square* tests for categorical variables and Student's *t*-tests for continuous variables.

	**Normally grown (n=29)**	**EUGR (n=22)**	**p-value**
	n (%)		
Male	13 (44.8)	9 (40.9)	0.780
Extremely premature (≤28 weeks)	6 (20.7)	6 (27.3)	0.583
Multiple pregnancy	7 (24.1)	7 (31.8)	0.543
Prenatal steroids (1 or more doses)	23 (79.3)	19 (86.4)	0.714
Mechanical ventilation	12 (41.4)	8 (36.4)	0.716
Non-invasive ventilation	26 (89.7)	21 (95.5)	0.625
Oxygen at 28 days of life	5 (17.2)	5 (22.7)	0.728
Oxygen 36 PMA	2 (6.9)	3 (13.6)	0.641
Patent Ductus Arteriosus	9 (31.0)	12 (54.5)	0.091
Surgical	2 (6.9)	1 (4.5)	1.000
Late Onset Sepsis	4 (13.8)	3 (13.6)	1.000
Retinopathy of Prematurity	4 (16.7)	6 (27.3)	0.484
Laser treated ROP	0 (0.0)	0 (0.0)	-
Necrotizing Enterocolitis	1 (3.4)	0 (0.0)	1.000
Surgical NEC	1 (3.4)	0 (0.0)	1.000
Intraventricular Hemorrhage	5 (17.2)	6 (27.3)	0.498
Severe IVH (grade III-IV)	2 (6.9)	2 (9.1)	1.000
	Mean (SD)		
Gestational age (weeks)	29.8 (1.8)	29.4 (1.9)	0.390
Postmenstrual age at discharge (weeks)	36.9 (2.0)	38.0 (2.1)	0.075
Length of stay (days)	49.7 (22.5)	60.0 (21.7)	0.103
Mechanical ventilation (days)	1.6 (4.0)	6.4 (24.0)	0.357
Oxygen therapy (days)	8.9 (19.1)	17.5 (34.4)	0.302
Antibiotic therapy (days)	5.5 (4.2)	10.1 (16.1)	0.149
Days on intensive care	4.4 (6.0)	11.4 (26.6)	0.175

Table S2. Differences in plasma amino acids and derivatives between well-grown and EUGR VPI.

Compound	ID HMDB	Statistical significance (q-value)				Percentage change between groups			
		EUGR *vs.* non-EUGR	EUGR-mod *vs.* non-EUGR	EUGR-sev *vs.* non-EUGR	EUGR-sev *vs.* EUGR-mod	EUGR *vs.* non-EUGR	EUGR-mod *vs.* non-EUGR	EUGR-sev *vs.* non-EUGR	EUGR-sev *vs.* EUGR-mod
L-Alanine	0000161	1.61E-02	ns	6.30E-03	*	-31	-17	-49	-39
L-Valine	0000883	3.08E-02	*	2.06E-02	*	-25	-15	-36	-25
L-Leucine/L-Isoleucine	0000687	2.10E-03	*	1.70E-03	ns	-28	-21	-37	-20
L-Phenylalanine	0000159	1.78E-02	*	1.46E-02	*	-22	-14	-31	-21
L-Tyrosine	0000158	4.65E-02	*	4.65E-02	ns	-28	-28	-29	-2
L-Tryptophan	0000929	*	ns	2.91E-02	2.91E-02	-7	8	-23	-29
L-Serine	0000187	*	ns	2.58E-02	*	-37	-27	-50	-31
L-Threonine	0000167	1.95E-02	ns	1.95E-02	ns	-28	-11	-47	-41
L-Asparagine	0000168	*	ns	*	ns	-20	-11	-31	-23
L-Glutamine	0000641	1.04E-02	ns	5.30E-03	*	-21	-12	-31	-22
L-Arginine	0000517	1.63E-02	ns	8.10E-03	*	-26	-14	-39	-29
L-Lysine	0000182	*	ns	*	*	-23	-13	-33	-22
L-Methionine	0000696	1.13E-02	*	7.80E-03	*	-36	-29	-45	-23
L-Cysteine	0000574	*	ns	*	*	-17	4	-42	-44
L-Cystine	0000192	*	ns	3.35E-02	*	-12	-2	-23	-21
Glycine	0000123	*	ns	*	*	-17	-10	-26	-17
L-Proline	0000162	3.18E-02	ns	5.50E-03	3.18E-02	-24	-7	-45	-41
L-Ornithine	0000214	7.20E-03	1.69E-02	1.69E-02	ns	-30	-30	-31	-3
N,N-dimetilglicine	0000092	*	ns	9.10E-03	9.10E-03	-18	3	-42	-44
Trans-4-hydroxyproline	0000725	*	ns	4.39E-02	*	-23	-10	-39	-33
N(6)-Methyllysine	0002038	3.72E-02	ns	3.27E-02	ns	-32	-16	-51	-42
N6,N6,N6-Trimethyl-L-lysine	0001325	4.10E-03	ns	3.50E-03	*	-18	-12	-26	-16
Homo-L-arginine	0000670	1.71E-02	ns	1.71E-02	ns	-20	-16	-24	-10
ADMA Dimethyl-L-Arginine	0003334	*	ns	2.95E-02	*	-9	-3	-17	-14
Glutamylglutamine	0028817	5.20E-03	ns	5.20E-03	ns	-41	-29	-55	-37
Glutamylleucine	0028823	4.50E-02	ns	1.60E-02	*	-18	-7	-29	-23
Pyroglutamic acid	0000267	1.07E-02	*	4.40E-03	*	-38	-28	-52	-33

ANOVA or Kruskal-Wallis pos-hoc p values for specified interpretations, EUGR *vs.* non-EUGR, EUGR-mod *vs.* non-EUGR, EUGR-sev *vs.* non-EUGR are reported as FDR corrected q values, with Q value set to 0.05. The direction of metabolite changes in EUGR, EUG-mod and EUGR-sev is expressed in percentage. Index “*”: significant difference in multivariate analysis with VIP>1.0 and p(corr) >0.5.

Table S3. Differences in plasma lipid species between well-grown and EUGR VPI.

Compound	Formula	Statistical significance (q-value)				Percentage change between groups			
		EUGR *vs.* non-EUGR	EUGR-mod *vs.* non-EUGR	EUGR-sev *vs.* non-EUGR	EUGR-sev *vs.* EUGR-mod	EUGR *vs.* non-EUGR	EUGR-mod *vs.* non-EUGR	EUGR-sev *vs.* non-EUGR	EUGR-sev *vs.* EUGR-mod
Glycerophospholipids									
LysoPC(14:0)	C22H46NO7P	1.79E-02	ns	2.60E-03	3.76E-02	-29	-18	-42	-29
LysoPC(15:0) or LysoPE(18:0)	C23H48NO7P	1.65E-02	ns	1.65E-02	ns	-29	-25	-33	-10
LysoPC(16:0) or LysoPE(19:0)	C24H50NO7P	2.12E-02	ns	1.17E-02	ns	-20	-13	-28	-18
LysoPC(O-16:0)	C24H52NO6P	4.30E-03	8.50E-03	2.12E-02	ns	-25	-26	-24	4
LysoPC(17:0) or LysoPE(20:0)	C25H52NO7P	4.88E-02	ns	2.25E-02	ns	-24	-18	-32	-17
LysoPC(18:0)	C26H54NO7P	3.60E-02	ns	1.56E-02	ns	-20	-13	-28	-17
LysoPC(O-18:0)	C26H56NO6P	2.39E-02	2.39E-02	ns	ns	-27	-29	-24	7
LysoPC(O-18:1) or LysoPC(P-18:0)	C26H54NO6P	8.30E-03	2.05E-02	2.05E-02	ns	-24	-23	-26	-4
LysoPC(19:0) or LysoPE(22:0)	C27H56NO7P	ns	ns	5.28E-02	ns	-19	-10	-30	-23
LysoPC(20:0)	C28H58NO7P	3.70E-03	ns	2.50E-03	ns	-22	-14	-32	-21
LysoPC(17:1) or LysoPE(20:1)	C25H50NO7P	4.72E-02	ns	ns	ns	-27	-25	-29	-6
LysoPE(18:1)	C23H46NO7P	3.12E-02	4.29E-02	4.29E-02	ns	-24	-24	-25	-2
LysoPC(18:1)	C26H52NO7P	1.40E-03	1.59E-02	1.70E-03	ns	-24	-20	-29	-11
LysoPC(20:1)	C28H56NO7P	1.67E-02	ns	1.67E-02	ns	-21	-16	-27	-13
LysoPC(18:2)	C26H50NO7P	3.30E-03	2.42E-02	3.90E-03	ns	-24	-20	-29	-11
LysoPC(19:3)	C27H50NO7P	1.67E-02	ns	1.67E-02	ns	-25	-20	-31	-14
LysoPC(20:5)	C28H48NO7P	ns	ns	3.06E-02	ns	-22	-3	-45	-43
LysoPC(20:2)	C28H54NO7P	3.60E-02	ns	3.60E-02	ns	-29	-23	-36	-17
LysoPC(20:3)	C28H52NO7P	1.07E-02	ns	1.07E-02	ns	-30	-22	-39	-22
LysoPC(20:4)	C28H50NO7P	1.91E-02	ns	1.91E-02	ns	-25	-20	-30	-12
LysoPE(20:4)	C25H44NO7P	2.46E-02	2.46E-02	ns	ns	-22	-24	-19	6
LysoPC(22:6)	C30H50NO7P	6.40E-03	ns	3.80E-03	ns	-27	-17	-40	-28
PC(31:1) or PE(34:1)	C39H76NO8P	ns	4.77E-02	ns	ns	-10	-14	-5	10
PC(32:0) or PE(35:0)	C40H82NO7P	ns	2.89E-02	ns	2.79E-02	-7	-18	7	30
PC(35:4) or PE(38:4)	C43H78NO8P	2.86E-02	ns	ns	ns	-22	-21	-22	0

Compound	Formula	Statistical significance (q-value)				Percentage change between groups			
		EUGR *vs.* non-EUGR	EUGR-mod *vs.* non-EUGR	EUGR-sev *vs.* non-EUGR	EUGR-sev *vs.* EUGR-mod	EUGR *vs.* non-EUGR	EUGR-mod *vs.* non-EUGR	EUGR-sev *vs.* non-EUGR	EUGR-sev *vs.* EUGR-mod
PC(P-36:0) or PC(O-36:1)	C44H88NO7P	8.80E-03	8.80E-03	ns	ns	-19	-24	-12	15
PC(38:4) or PE(41:4)	C46H84NO8P	ns	ns	1.91E-02	ns	-24	-11	-40	-33
PC(39:4) or PE(42:4)	C47H86NO8P	2.10E-03	ns	1.90E-03	ns	-28	-19	-39	-25
PC(39:6)	C47H82NO8P	ns	ns	3.07E-02	ns	-15	-2	-31	-30
PC(39:7) or PE(42:7)	C47H80NO8P	8.50E-03	ns	8.50E-03	ns	-34	-24	-45	-28
PC(40:8)	C48H80NO8P	2.49E-02	ns	8.70E-03	ns	-18	-12	-25	-15
PC(42:6)	C50H88NO8P	8.60E-03	ns	8.60E-03	ns	-18	-14	-22	-10
PI(37:5)	C46H79O13P	7.20E-03	1.39E-02	2.68E-02	ns	-18	-19	-17	2
PI(40:4)	C49H87O13P	1.30E-03	1.30E-03	ns	ns	-35	-43	-25	33
Glycerophosphocholine	C8H20NO6P	2.14E-02	3.54E-02	3.54E-02	ns	-51	-50	-53	-6
Glycerolipids									
TG(52:3)	C55H100O6	4.64E-02	4.64E-02	ns	ns	16	23	7	-13
TG(57:3)	C60H110O6	2.23E-02	2.23E-02	ns	ns	19	25	11	-11
TG(52:4)	C55H98O6	2.40E-02	2.76E-02	ns	ns	24	28	19	-7
TG(57:4)	C60H108O6	1.03E-02	2.32E-02	2.32E-02	ns	28	32	23	-6
TG(54:6)	C57H98O6	2.86E-02	2.99E-02	ns	ns	19	24	13	-9
Sphingolipids									
SM(36:0)	C41H85N2O6P	3.38E-02	3.38E-02	ns	ns	-26	-29	-22	10
SM(38:0)	C43H89N2O6P	1.20E-03	3.90E-03	1.11E-02	ns	-33	-34	-32	4
SM(40:0)	C45H93N2O6P	1.78E-02	3.02E-02	3.02E-02	ns	-38	-37	-39	-3
SM(41:0)	C46H95N2O6P	1.20E-03	1.84E-02	1.40E-03	ns	-47	-42	-51	-16
SM(42:0)	C47H97N2O6P	8.50E-03	1.04E-02	ns	ns	-42	-45	-40	9
SM(32:1)	C37H75N2O6P	2.31E-02	ns	2.31E-02	ns	-19	-14	-25	-12
SM(33:1)	C38H77N2O6P	1.49E-02	1.49E-02	ns	ns	-14	-15	-13	2
SM(34:1)	C39H79N2O6P	1.84E-02	1.84E-02	ns	ns	-11	-14	-9	6
SM(35:1)	C40H81N2O6P	3.91E-02	ns	4.40E-02	ns	-24	-21	-27	-8
SM(37:1)	C42H85N2O6P	1.70E-03	6.30E-03	7.80E-03	ns	-19	-20	-19	0
SM(38:1)	C43H87N2O6P	4.50E-03	ns	4.50E-03	ns	-34	-30	-39	-13

Compound	Formula	Statistical significance (q-value)				Percentage change between groups			
		EUGR *vs.* non-EUGR	EUGR-mod *vs.* non-EUGR	EUGR-sev *vs.* non-EUGR	EUGR-sev *vs.* EUGR-mod	EUGR *vs.* non-EUGR	EUGR-mod *vs.* non-EUGR	EUGR-sev *vs.* non-EUGR	EUGR-sev *vs.* EUGR-mod
SM(39:1)	C44H89N2O6P	3.20E-03	1.80E-02	5.70E-03	ns	-24	-21	-27	-8
SM(40:1)	C45H91N2O6P	1.14E-02	3.50E-02	1.76E-02	ns	-17	-15	-19	-5
SM(41:1)	C46H93N2O6P	1.11E-02	4.13E-02	1.36E-02	ns	-22	-19	-25	-8
SM(42:1)	C47H96N2O6P	2.70E-02	3.73E-02	ns	ns	-30	-35	-24	18
SM(43:1)	C48H97N2O6P	3.10E-03	8.20E-03	1.79E-02	ns	-45	-43	-49	-10
SM(38:2)	C43H83N2O6P	1.48E-02	1.48E-02	ns	ns	-25	-29	-20	13
SM(41:2)	C46H91N2O6P	2.26E-02	1.67E-02	ns	ns	-17	-23	-9	19
SM(43:2)	C48H95N2O6P	2.70E-02	4.68E-02	4.68E-02	ns	-42	-43	-41	4
SM(44:2)	C49H97N2O6P	2.70E-03	6.50E-03	1.89E-02	ns	-28	-32	-22	15
Cer(40:0)	C40H81NO3	2.40E-03	8.40E-03	8.40E-03	ns	-22	-21	-24	-3
Cer(41:0)	C41H83NO3	<0.0001	<0.0001	<0.0001	ns	-34	-33	-36	-5
Cer(42:0)	C42H85NO3	1.00E-04	1.10E-03	1.70E-03	ns	-34	-34	-34	1
Cer(40:1)	C40H79NO3	1.11E-02	ns	1.11E-02	ns	-18	-15	-22	-8
Cer(41:1)	C41H81NO3	4.60E-03	1.93E-02	9.00E-03	ns	-24	-21	-27	-7
Cer(42:1)	C42H83NO3	1.17E-02	2.80E-02	2.80E-02	ns	-31	-31	-30	1
Cer(44:2)	C44H85NO3	8.40E-03	8.40E-03	ns	ns	-26	-29	-21	11
Cer(43:1)	C43H85NO3	2.00E-04	2.20E-03	7.00E-04	ns	-22	-20	-25	-6
Other lipids									
Taurocholic acid	C26H45NO7S	ns	ns	8.10E-03	1.30E-03	37	-35	123	246
Taurochenodeoxycholic acid or Taurodeoxycholic acid	C26H45NO6S	ns	ns	*	*	36	-40	128	283
Glycochenodeoxycholate-3-sulfate	C26H43NO8S	3.99E-02	ns	3.99E-02	*	37	10	68	53
Glycocholic acid	C26H43NO6	ns	ns	1.52E-02	ns	48	11	93	74
Tetrahydroxycholestanoic acid	C27H46O6	ns	ns	7.10E-03	7.00E-04	30	-31	103	194
Cholic acid glucuronide	C30H48O11	ns	ns	1.17E-02	*	51	0	113	114
Hydroxycholesterol sulfate	C27H46O5S	ns	ns	4.60E-03	1.20E-03	2	-32	42	110
Oleic acid	C18H34O2	ns	2.62E-02	ns	2.45E-02	-13	-36	14	78
Linoleic acid	C18H32O2	ns	*	2.04E-02	*	-10	-28	11	53

Compound	Formula	Statistical significance (q-value)				Percentage change between groups			
		EUGR *vs.* non-EUGR	EUGR-mod *vs.* non-EUGR	EUGR-sev *vs.* non-EUGR	EUGR-sev *vs.* EUGR-mod	EUGR *vs.* non-EUGR	EUGR-mod *vs.* non-EUGR	EUGR-sev *vs.* non-EUGR	EUGR-sev *vs.* EUGR-mod
Palmitic acid	C16H32O2	ns	3.10E-02	ns	3.31E-02	-10	-21	4	31
Arachidonic acid	C20H32O2	*	3.05E-02	ns	3.05E-02	-13	-36	15	80
Hydroxypalmitic acid	C16H32O3	4.30E-03	1.70E-03	ns	ns	-19	-27	-10	24
Linoleoyl carnitine	C25H45NO4	3.61E-02	3.61E-02	ns	ns	-23	-29	-15	20
Oleoylcarnitine	C25H47NO4	3.37E-02	3.37E-02	ns	ns	-24	-30	-16	21
Palmitoylcarnitine	C23H45NO4	3.99E-02	3.99E-02	ns	ns	-19	-26	-11	20
Stearoylcarnitine	C25H49NO4	3.56E-02	4.56E-02	ns	ns	-24	-25	-22	3

ANOVA or Kruskal-Wallis pos-hoc p values for specified interpretations, EUGR *vs.* non-EUGR, EUGR-mod *vs.* non-EUGR, EUGR-sev *vs.* non-EUGR are reported as FDR corrected q values, with Q value set to 0.05. The direction of metabolite changes in EUGR, EUG-mod and EUGR-sev is expressed in percentage. Index “*”: significant difference in multivariate analysis with VIP>1.0 and p(corr) >0.5.

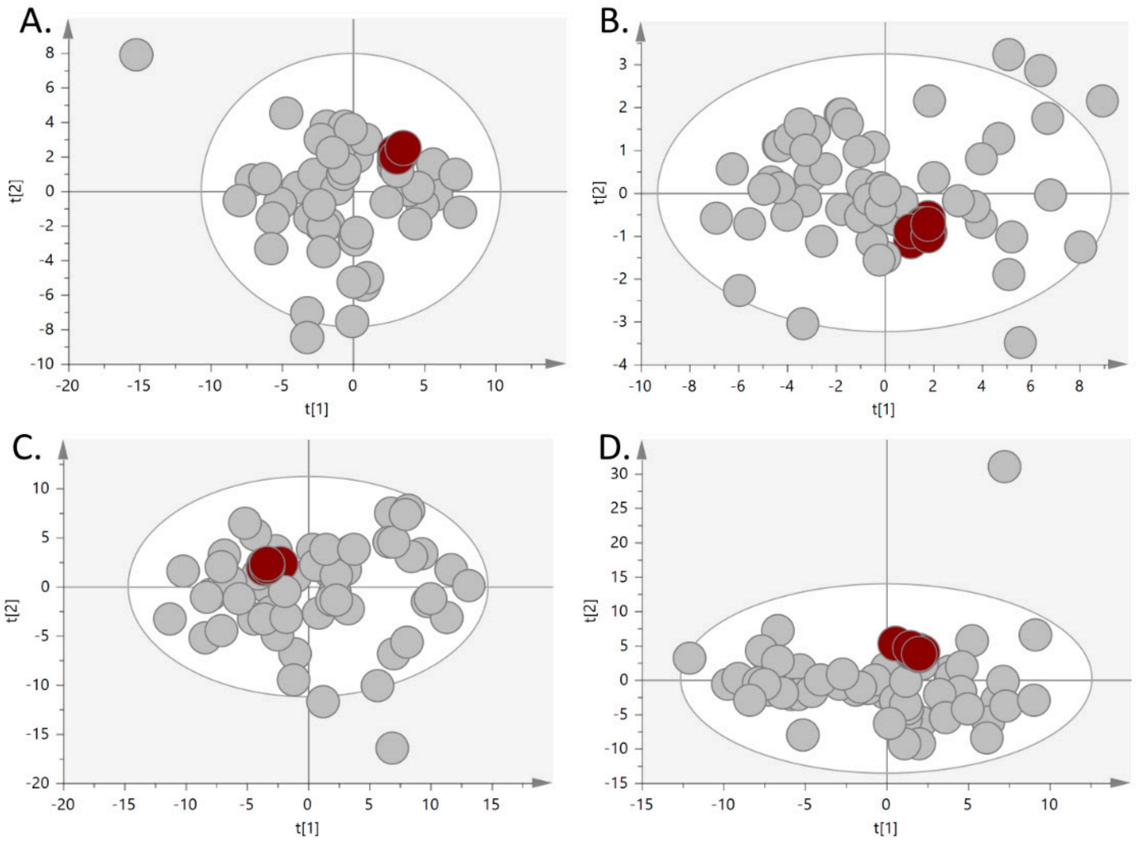

Supplementary Figure 1. PCA-X model for the prediction of pooled QC samples (dark red) analyzed throughout the entire sequence in (A) CE-MS, (B) GC-MS, (C) LC-MS/ESI+ and (D) LC-MS/ESI-. Data show very good clustering, thereby indicating good system stability and reliability of the results. The observations outside the ellipse defined by Hotelling´s T2 (with 95% confidence level) were considered as outliers. .

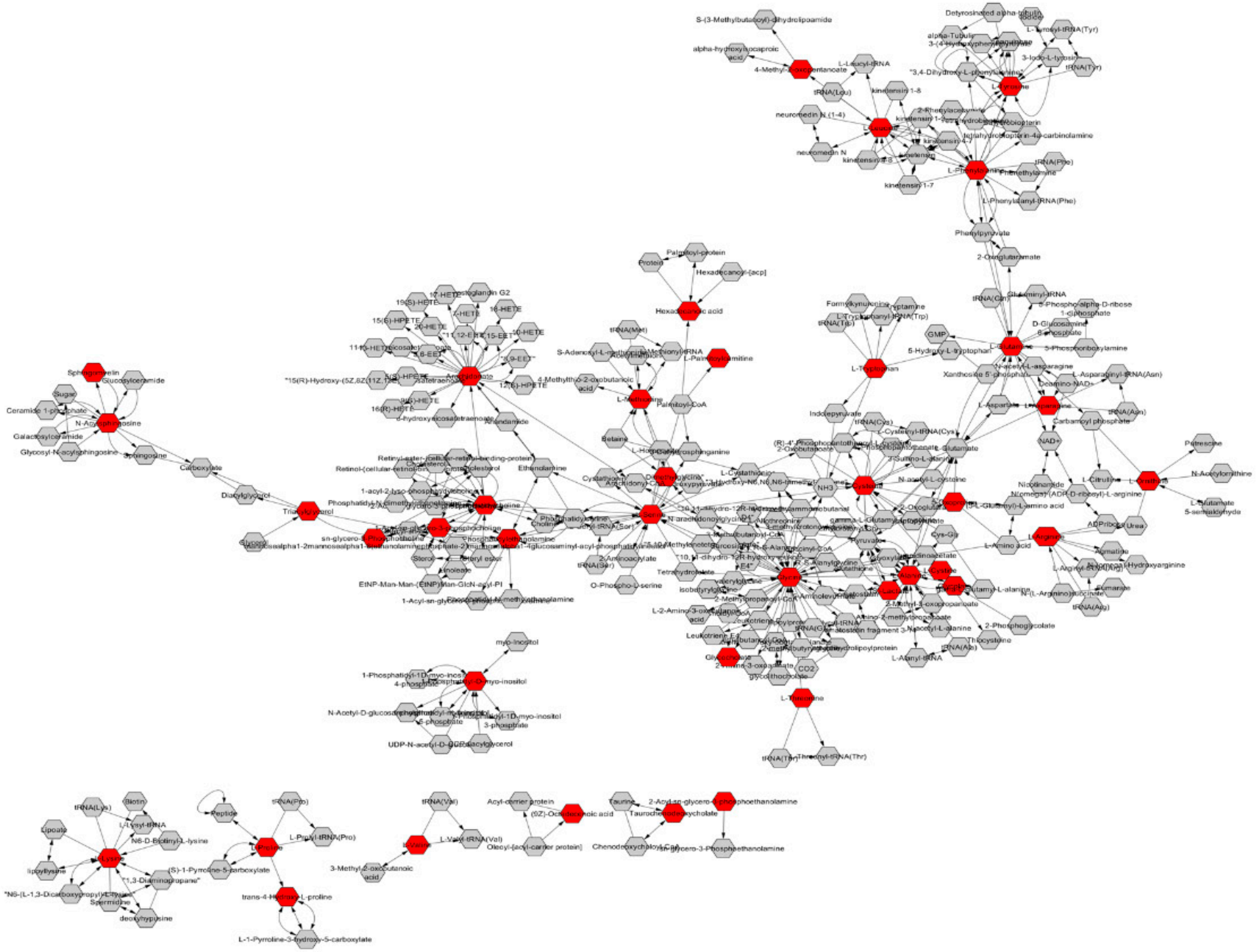

Supplementary Figure 2. Visual representation of metabolomics data consisting of the network of metabolites involved in EUGR process based on KEGG IDs reported for statistically significant compounds. Lipid species were mapped by lipid class KEGG IDs. The red nodes represent mapped metabolites and edges represent related biochemical reactions.
